# DLX2 Is a Potential Immune-Related Prognostic Indicator Associated with Remodeling of Tumor Microenvironment in Lung Squamous Cell Carcinoma: An Integrated Bioinformatical Analysis

**DOI:** 10.1155/2022/6512300

**Published:** 2022-10-21

**Authors:** Liling Huang, Tongji Xie, Fuqiang Zhao, Yu Feng, Haohua Zhu, Le Tang, Xiaohong Han, Yuankai Shi

**Affiliations:** ^1^Department of Medical Oncology, National Cancer Center/National Clinical Research Center for Cancer/Cancer Hospital, Chinese Academy of Medical Sciences & Peking Union Medical College, Beijing Key Laboratory of Clinical Study on Anticancer Molecular Targeted Drugs, Beijing 100021, China; ^2^Department of Colorectal Surgery, National Cancer Center/National Clinical Research Center for Cancer/Cancer Hospital, Chinese Academy of Medical Sciences & Peking Union Medical College, Beijing 100021, China; ^3^Clinical Pharmacology Research Center, Peking Union Medical College Hospital, State Key Laboratory of Complex Severe and Rare Diseases, NMPA Key Laboratory for Clinical Research and Evaluation of Drug, Beijing Key Laboratory of Clinical PK & PD Investigation for Innovative Drugs, Chinese Academy of Medical Sciences & Peking Union Medical College, Beijing 100730, China

## Abstract

**Background:**

It is still an unmet clinical need to identify potent biomarkers for immunotherapy on patients with lung squamous cell carcinoma (LUSC).

**Methods:**

In this study, we explored the differentially expressed genes (DEGs) that were simultaneously correlated with four pathways (i.e. CD8^+^*αβ*T cell proliferation/differentiation/activation pathways and ferroptosis pathway) and possibly related to the remodeling of tumor microenvironment via the TCGA-LUSC dataset. Besides, four GEO datasets (GSE157009, GSE157010, GSE19188, and GSE126045) and IMvigor210 dataset were utilized for confirmation and validation.

**Results:**

The co-downregulated DEG DLX2 was selected for further analysis. Function enrichment analysis revealed that low-expression of DLX2 was closely related to various immune-related pathways like T/B/NK cell mediated immunity, interferon gamma/alpha response, and various autoimmune disease. DLX2-downregulated group was enriched in more immune-activating cells and lower tumor immune dysfunction and exclusion (TIDE) score. Via the Cancer Immunome Atlas (TCIA) database, lower expression of DLX2 was also found to be associated with better IPS score of PD-1/PD-L1 blockade (*p* < 0.001) as well as CTLA-4 combined with PD-1/PD-L1 blockade (*p* < 0.001). Furthermore, patients in DLX2-low group were found to have significant longer median OS than those in DLX2-high group in IMvigor210 dataset (10.8 vs 7.4 months; hazard ratio [HR]=0.74, 95% confidence interval [95%CI] 0.57-0.96; *p* = 0.024).

**Conclusions:**

Our study on an integrated bioinformatical analysis implied that DLX2 could be served as a promising indicator for remodeling tumor microenvironment status and predicting ICI response of patients with LUSC.

## 1. Introduction

According to GLOBOCAN 2020, lung cancer is one of the most frequently diagnosed cancers and the leading cause of cancer-related deaths worldwide [1]. Lung squamous cell carcinoma (LUSC) is a common histological subtype of non-small cell lung cancer (NSCLC), which accounts for 17% [2]. In recent years, immunotherapy by immune checkpoint inhibitors (ICIs) have brought about a revolutionary shift in the treatment strategies of LUSC with significantly improved clinical outcome, while limited patients can achieve durable tumor response and long term survival. All of the existing biomarkers like programmed cell death-ligand 1 (PD-L1) have unsatisfying prognostic value, it is still an unmet clinical need to find novel and effective biomarkers for precise immunotherapy [3–7].

The immunotherapy via programmed cell death protein 1 (PD-1)/PD-L1 pathway blockade largely relies on the infiltration of efficient T cells into tumor and the activity of effector T cells in the tumor microenvironment (TME) [8]. *αβ*T cell is a predominant subtype of T cell which account for about 95% [9]. During the treatment of immunotherapy, CD8^+^ T cells are activated and extinguish tumor cells mainly through perforin-granzyme-based and Fas-based mechanisms [10]. Ferroptosis is emerged as a form of regulated cell death featured by the iron-dependent accumulation of lipid hydroperoxides [11, 12]. Recently, ferroptosis has attracted certain attention in antitumor research area. A recent finding proposed that tumor ferroptosis promoted by immunotherapy-activated CD8^+^ T cell is another mechanism to enhance antitumor efficacy of immunotherapy [13]. The activity of signal pathways related to CD8^+^ T cell and ferroptosis process may better reflect the human immune status and help us detect promising prognostic biomarkers.

This study aimed to determine biomarkers associating with the remodeling of tumor microenvironment and predicting efficacy of ICIs therapy in patients with LUSC. We firstly explored targeted genes that are simultaneously correlated with four pathways including CD8^+^*αβ*T cell proliferation/differentiation/activation pathways and ferroptosis pathway using the bioinformatics technology, then focused on the co-downregulated differentially expressed genes (DEGs) Distal-Less Homeobox 2 (DLX2), which acted as a transcriptional activator. Its correlation with immune cells of TME, clinicopathological factors as well as its value to predict the efficacy of immunotherapy were evaluated deeply, aiming to facilitate personalized immunotherapy of LUSC.

## 2. Materials and Method

### 2.1. Data Collection

491 somatic mutation information (simple nucleotide variation [SNV]), 501 transcriptome profiling (HTSeq-FPKM of mRNA) and 498 cases with clinical data of patients with LUSC were achieved from Genomic Data Commons (GDC) Data Portal of the Cancer Genome Atlas (TCGA) (https://portal.gdc.cancer.gov) and then a total of 482 LUSC tumor samples with complete SNV, mRNA, and clinical data were included for the detection of targeted genes and their characteristics and functions. Besides, through the Gene Expression Omnibus (GEO) database (https://www.ncbi.nlm.nih.gov/geo), we also selected three cohorts with gene expression and clinical data of LUSC patients (GSE157009, GSE157010, and GSE19188) for confirmation and validation [14, 15]. 9 patients with LUSC receiving the treatment of anti-PD-1 antibody nivolumab was obtained from GSE126045 for clinical validation [16]. Besides, another IMvigor210 dataset of 348 patients with urothelial carcinoma receiving the treatment of anti-PD-L1 antibody atezolizumab was used to expand our validation cohort and to investigate the prognostic value of DLX2 on ICI treatment [17]. The detailed information about the six datasets included in this analysis were summarized in Supplementary table 1.

### 2.2. Estimate of CD8^+^ T Cell Related Pathway Score and Ferroptosis Score

We employed three pathways from Molecular Signatures Database (MSigDB) to evaluate the relative activity of CD8^+^*αβ*T cell differentiation and proliferation, and activation (Supplementary table 2). Besides, 60 ferroptosis-related genes were retrieved to estimate the ferroptosis score [11, 12, 18, 19]. “GSVA” package [20] was used to calculate scores for 482 patients of TCGA-LUSC dataset in each four pathways. In each pathway, patients were divided into high-score (higher than median score) or low-score (lower than or equal to median score) group by comparing to the median score (cut-off value).

### 2.3. Identification of Target DEGs

In each pathway, DEGs were identified by comparing the expression of every mRNA between high-score and low-score group through wilcoxon rank-sum test with the following criteria: (i). |log2 fold change(FC)| > 1, (ii). false discovery rate (FDR) < 0.25, (iii) *p* < 0.05. FC was defined as the ratio of median gene expression between high and low score group. The upregulated and downregulated DEGs were shown through heat map using “pheatmap” package and volcano plot using “ggplot2” package. Then intersection of upregulated DEGs or downregulated DEGs in the four pathways were performed to screen targeted DEGs. Next, TCGA and other validation datasets were divided into high-expression and low-expression groups for further analysis according the median value of targeted DEG in each dataset. The correlation between DEGs expression and scores of four pathways were evaluated by spearman method and presented in circos plot.

### 2.4. Somatic Mutation Data Analysis

The somatic mutation information of TCGA-LUSC samples was used in genomic analysis, which was processed by VarScan to remove germline mutation and loss of heterozygosity (LOH). We used “maftools” package [21] to present top 30 genes with the highest incidence of mutation by oncoplot.

### 2.5. Clinicopathological Characteristics Analysis

The correlation of targeted DEGs with clinicopathological characteristics: samples (tumor vs normal paracancerous tissue), age (≤65 vs >65 years), gender (male vs. female), TNM stage were evaluated by Wilcoxon rank sum test or Kruskal-Wallis test, the results were shown by boxplot via “ggpubr” package. The *p* value of pairwise comparison after Kruskal-Wallis test was adjusted by method of “holm”. The survival analysis was performed by log-rank test and Kaplan-Meier plot using the “survival” and “survminer” packages.

### 2.6. Function Enrichment Analysis

Gene Ontology biological process (GOBP), Kyoto Encyclopedia of Genes and Genomes (KEGG), and Hallmark database were used to evaluate relative activity of pathways among LUSC patients via two methods: Gene Set Enrichment Analysis (GSEA) and gene set variation analysis (GSVA). GSEA was performed using GSEA software, GSVA score was calculated by “GSVA” package, and wilcoxon rank-sum test was used to compare the scores between high and low targeted gene expression groups. The results were shown in balloonplot by “ggplot2” and “ggpubr” package.

### 2.7. Tumor-Infiltrating Immune Cells Analysis

QuanTIseq and single sample GSEA (ssGSEA) were utilized to estimate the abundance profile of tumor-infiltrating immune cells (TICs) in LUSC cases through “immunedeconv” [22] and “GSVA” package, respectively. Wilcoxon rank-sum test and Spearman correlation test were used to explore the relationship between targeted DEGs and immune cells in differential analysis and correlation analysis, respectively. Results of differential analysis were shown in boxplot while correlation analysis was in balloonplot, which were both via “ggpubr” package.

### 2.8. Efficacy Prediction of Immunotherapy

ImmuneScore (defined as proportion of immune ingredient), StromalScore (defined as proportion of stromal ingredient), and ESTIMATEScore (a symbol of tumor purity, defined as sum of ImmuneScore and StromalScore) of each LUSC sample were calculated using “estimate” package [23]. The higher score indicated higher ratio of immune/stromal component in the TME. We used mRNA expression level of CD274 to represent the expression of PD-L1. Besides, via the Cancer Immunome Atlas (TCIA) (https://tcia.at) [24], the association between the expression of targeted DEGs and immunophenoscore (IPS) was explored to predicting its value on predicting the efficacy of immunotherapy (i.e. PD-1/PD-L1 blockade and/or cytotoxic T-lymphocyte-associated antigen 4 [CTLA-4] blockade) on TCGA-LUSC patients. Tumor immune dysfunction and exclusion (TIDE) algorithm was also used to predict response of ICI treatment [25]. Finally, data of IMvigor210 and GSE126045 datasets were obtained for clinical validation.

### 2.9. Statistical Analysis

All statistical analyses were performed by R software (version ≥3.6.2; https://www.r-project.org) and GSEA software (version: 4.0.3, https://www.broadinstitute.org/gsea). *p* value < 0.05 was considered statistically significant.

## 3. Results

### 3.1. Analysis Workflow of the Study

**Figure 1:**
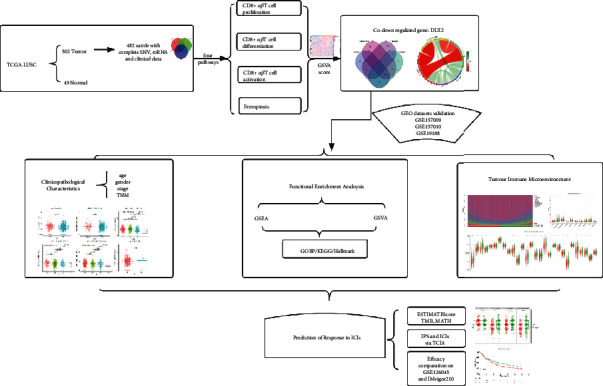
Study design and overview workflow.

**Figure 2:**
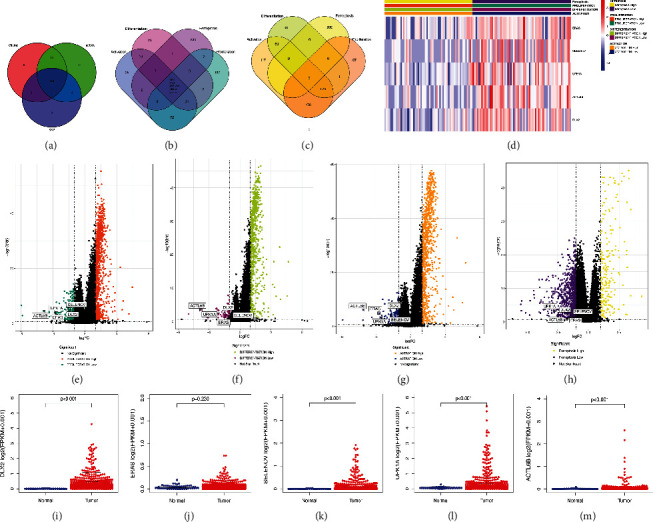
Identification of DEGs in four pathways. (a) Venn plot showing 482 samples were derived with completed SNV, mRNA, and clinical data. (b) Venn plot showing co-downregulated DEGs. (c) Venn plot showing co-upregulated DEGs. (d) Heat map visualizing the identification of DEGs in the four pathways. (e–h) Volcano plots of four pathways: CD8^+^*αβ*T cell proliferation pathway (e), CD8^+^*αβ*T cell differentiation pathway (f), CD8^+^*αβ*T cell activation pathway (g), and ferroptosis pathway (h). (i–m) The comparison of DEGs expression in tumor samples and normal paracancerous samples: DLX2 (i), ERAS (j), SELENOV (k), UPK1A (l), and ACTL6B (m). (DEG, differentially expressed genes; SNV, simple nucleotide variation).

In order to investigate potential immune-related genes that are associated with remodeling of tumor microenvironment in LUSC and capable of predicting efficacy of immunotherapy, this study was carried out according to the following analysis process (Figure 1). A total of 482 TCGA-LUSC patients with complete SNV, mRNA, and clinical data were included (Figure 2(a)), then the score of four pathways (CD8^+^*αβ*T cell proliferation, CD8^+^*αβ*T cell differentiation, CD8^+^*αβ*T cell activation, and ferroptosis pathways) in these patients were calculated. DEGs were identified by comparing the expression of every gene between high-score and low-score group, the co-upregulated or co-downregulated genes were selected as candidate targeted DEGs. By intersection, 5 co-downregulated DEGs DLX2, ERAS, SELENOV, UPK1A, and ACTL6B were identified, while no co-upregulated DEG was found. Then we focused on DLX2 for further analysis, including clinicopathological characteristics, function enrichment analysis, TICs analysis, efficacy of ICIs treatment, etc. Besides, four GEO datasets (GSE157009, GSE157010, GSE19188, and GSE126045) and IMvigor210 dataset were utilized for validation.

### 3.2. Identification of DLX2 as a Co-downregulated DEG in the Four Pathways

The clinicopathological characteristics of TCGA-LUSC patients were summarized in Supplementary Table 3. 863 upregulated DEGs and 223 downregulated DEGs were obtained from score of CD8^+^*αβ*T cell proliferation pathway. 678 upregulated DEGs and 60 downregulated DEGs were obtained from score of CD8^+^*αβ*T cell differentiation pathway. 932 upregulated DEGs and 146 downregulated DEGs were obtained from score of CD8^+^*αβ*T cell activation pathway. 202 upregulated DEGs and 855 downregulated DEGs were obtained from score of ferroptosis pathway. The intersection of DEGs was visualized in a Venn diagram (Figures 2(b) and 2(c)). The heat map of the five co-downregulated DEGs was shown in Figure 2(d). The volcano plots of four pathways were presented in Figures 2(e)–2(h). The differential analysis results of five DEGs in the four pathways were summarized in Supplementary Table 4. We also explored the expression profiles of the five downregulated DEGs in tumor samples compared to normal paracancerous ones. Except for ERAS, other four DEGs were significantly highly expressed in tumor samples (Figures 2(i)–2(m)).

After exploring and comparing of the five DEGs, low expression of DLX2 was found to have the closest association with various immune-related pathways, larger amount of immune-activating infiltration cells, and IPS of PD-1/PD-L1 blockade and/or combined with CTLA-4 blockade, which might exert impact on enhancing the immunotherapeutic response of LUSC. Hereon we focused on DLX2 in this study. All TCGA samples were divided into DLX2-high and DLX2-low group by comparing to the median expression value of DLX2 (FPKM value = 0.152, *z* − score = 0.1044).

**Figure 3:**
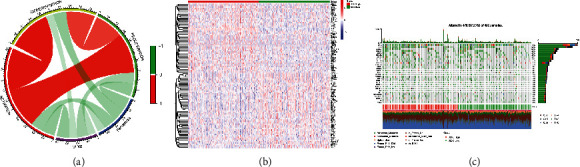
Relationship between DLX2 and four pathways. (a) Circos plot showing the correlation between DLX2 expression and four pathways. (b) Heat map visualizing the identification of DEGs conducted by comparing high DLX2 and low DLX2 expression group. (c) Waterfall plot presenting the overview of somatic mutations in all TCGA-LUSC samples.

The correlation between DLX2 expression and scores of four pathways were presented in circos plot (Figure 3(a)), which showed that DLX2 expression was negatively correlated with scores of all these four pathways. Besides score of ferroptosis pathway presented a negative correlation with the other three CD8^+^*αβ*T cell-related pathways. The DEGs screened by expression of DLX2 was presented in Figure 3(b). In addition, the detailed information of the top 30 most frequently mutated genes of all 482 TCGA-LUSC samples divided by DLX2 expression was presented in oncoplot (Figure 3(c)).

### 3.3. Expression of DLX2 Remains Stable in Different Clinicopathological Characteristics Subgroups

We attempted to investigate the relationship between the expression of DLX2 and the clinicopathological characteristics including age (≤65 vs >65 years), gender (male vs female), TNM stages of patients in TCGA-LUSC dataset. Interestingly, no significant differences were observed in any clinicopathological characteristics we included (Supplementary figure 1, age: *p* = 0.95; gender: *p* = 0.56; TNM stage: *p* = 0.50; T stage: *p* = 0.13; N stage: *p* = 0.19; M stage: *p* = 0.26). The above results indicated that the proportion of expression of DLX2 might be similar in different TNM stage, age, and gender of LUSC patients. Besides expression of DLX2 was not a candidate prognostic indicator for overall survival of patients with LUSC (*p* = 0.972). The results were consistent with those found in three GEO datasets (i.e, GSE157009, GSE157010, and GSE19188). These results showed that the expression of DLX2 remained stable in different clinicopathological characteristics subgroups of patients with LUSC.

### 3.4. Functional Enrichment Analysis

**Figure 4:**
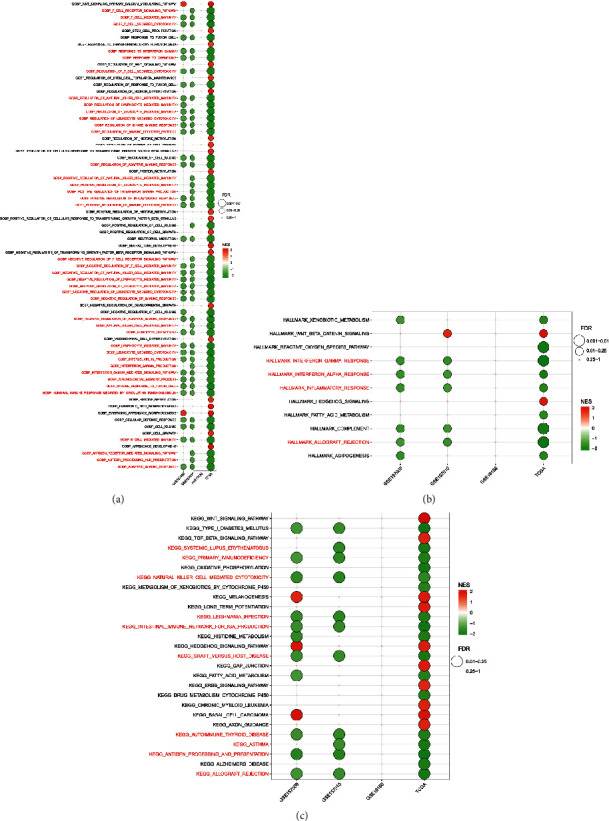
Functional enrichment analysis of DLX2 by GSEA. (a) GOBP enrichment analysis. (b) Hallmark enrichment analysis. (c) KEGG enrichment analysis.

GOBP, KEGG, and hallmark enrichment analysis were applied by GSEA to evaluate the biological function of DLX2 (Figure 4). GOBP enrichment analysis revealed that lower expression of DLX2 was closely associated with T/B cell mediated immunity, T-cell mediated cytotoxicity, regulation of natural killer cell/lymphocyte/leukocyte mediated immunity, positive regulation of immune effector process, adaptive immune response, antigen processing, and presentation. While higher expression of DLX2 was associated with embryonic digit morphogenesis, cell growth, neural tube development, regulation of cellular response to transforming growth factor beta stimulus, regulation of histone methylation, and protein methylation. KEGG enrichment analysis revealed that DLX2-low group was associated with systemic lupus erythematosus (SLE), graft versus host disease, autoimmune thyroid disease, and other immune-related disease. DLX2-high group was associated with Wnt signaling pathway, ERBB signaling pathway, basal cell carcinoma, etc. Hallmark enrichment analysis revealed that interferon gamma response, interferon alpha response, and inflammatory response were activated in DLX2-low group, meanwhile DLX2-high group is associated with Wnt signaling pathway. GOBP, KEGG, and hallmark enrichment analysis were also conducted by GSVA with similar results (Supplementary figure 2). Together, results of functional enrichment analysis strongly supported the correlation between low DLX2 expression with immune-related function.

### 3.5. Correlation of DLX2 Expression with Tumor-Infiltrated Immune Cells

**Figure 5:**
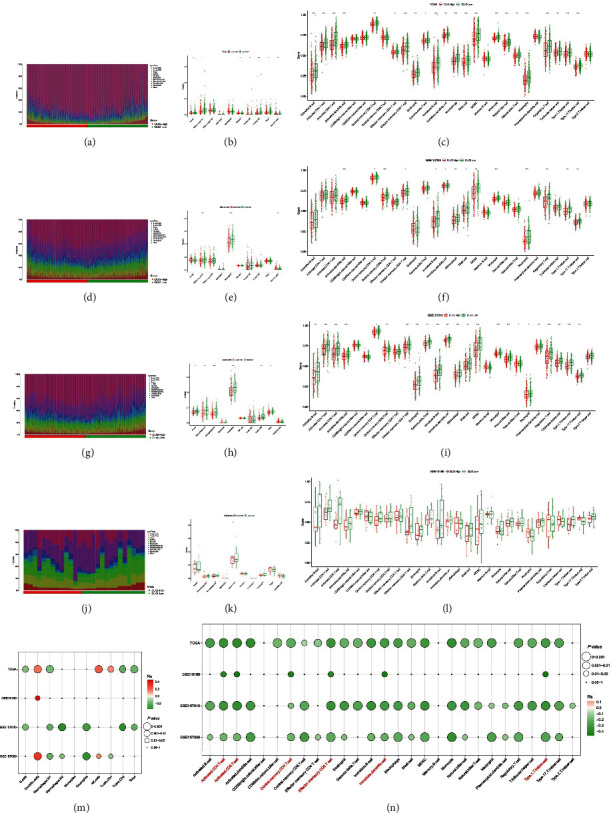
The abundance of TILs in high DLX2-expression group and low DLX2-expression group. Results of TCGA-LUSC data set by quanTIseq (a, b) and ssGSEA (c). Results of GSE157009 dataset by quanTIseq (d, e) and ssGSEA (f). Results of GSE157010 dataset by quanTIseq (g, h) and ssGSEA (i). Results of GSE19188 data set by quanTIseq (j, k) and ssGSEA (l). Bubble plots comprehensively representing the correlation of DLX2 expression and abundance of immune cells infiltration in all four datasets by quanTIseq (m) and ssGSEA (n). The asterisks indicated the statistical *p* value (^∗^: 0.01 < *p* < 0.05; ^∗∗^: 0.01 < *p* < 0.001; ^∗∗∗^: *p* < 0.001). (TIL, tumor-infiltrating lymphocytes; ssGSEA, single sample gene set enrichment analysis; TCGA, the Cancer Genome Atlas; LUSC, lung squamous cell carcinoma).

To further investigate the correlation between DLX2 and the TME, we applied quanTIseq and ssGSEA algorithm to provide an insight into the differential analysis between the expression of DLX2 and the abundance of various types of immune cells of patients with LUSC in TCGA (Figures 5(a)–5(c)), GSE157009 (Figures 5(d)–5(f)), GSE157010 (Figures 5(g)–5(i)), and GSE19188 (Figures 5(j)–5(l)) datasets. Moreover, the correlation of DLX2 expression and abundance of immune cells infiltration in the four datasets were comprehensively summarized and capable for comparison by balloonplots (Figure 5(m) for quanTIseq, Figure 5(n) for ssGSEA). Patients with high DLX2 expression have lower infiltration of activated CD4 T cells, activated CD8 T cells, central memory CD4 T cells, effector memory CD8 T cells, immature dentritic cells, and type 1 T helper cells in all of the four datasets. Besides, others immune-related cells like macrophages M1 are enriched in DLX2-low subgroup in most datasets.

### 3.6. DLX2 as a Promising Prognostic Predictor for ICI treatment

**Figure 6:**
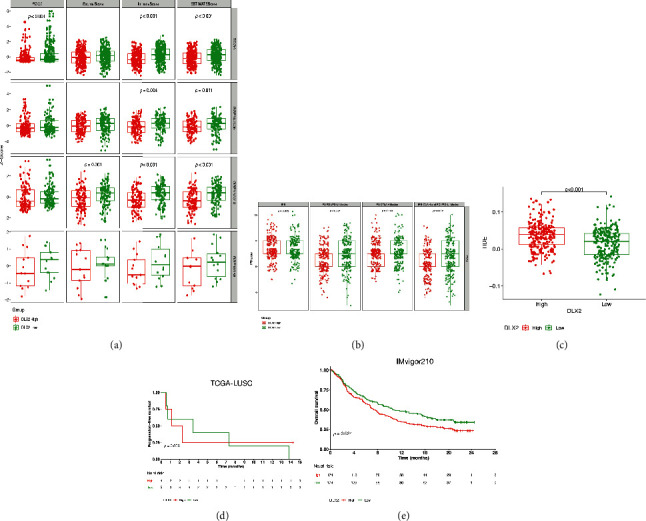
Prognostic value of DLX2 on immunotherapy. (a) The correlation between DLX2 expression and PD-L1, StromalScore, immuneScore, and EstimateScore in TCGA-LUSC and other GEO data sets. (b) The correlation between DLX2 expression and IPS, IPS of PD-1/PD-L1 blocker, IPS of CTLA4 blocker, as well as IPS of CTLA4 and PD-1/PD-L1 blocker via TCIA website. (c) the correlation between DLX2 and TIDE in TCGA-LUSC dataset. (d) Kaplan-Meier curve of PFS for patients with DLX2-low and DLX2-high groups in TCGA-LUSC dataset. (e) Kaplan-Meier curve of OS for patients with DLX2-low and DLX2-high groups in IMvigor210 dataset. (TCGA, the Cancer Genome Atlas; LUSC, lung squamous cell carcinoma; GEO, the Gene Expression Omnibus; IPS, immunophenoscore; TCIA, the Cancer Immunome Atlas; PD-1, programmed cell death protein 1; PD-L1, Programmed cell death-ligand 1; CTLA4, cytotoxic T-lymphocyte-associated antigen 4; TIDE, tumour immune dysfunction and exclusion).

In TCGA-LUSC dataset, the level of PD-L1 expression, ImmuneScore, and ESTIMATEScore were significantly higher in DLX2-low subgroup (*p* < 0.001 each). In validation of other three GEO datasets, though the significance varied, consistent tendency was observed (Figure 6(a)). The results indicated that low-DLX2 expression was correlated with a more hot-tumor environment and might promote the activity of immunotherapy.

Via TCIA website, lower expression of DLX2 was significantly associated with better PD-1/PD-L1 blockade (*p* < 0.001) as well as CTLA-4 combined and PD-1/PD-L1 blockade (*p* < 0.001), while expression of DLX2 seems had no correlation with IPS (*p* = 0.872) and CTLA-4 blockade (*p* = 0.169) (Figure 6(b)). Together, lower expression of DLX2 was more likely to be associated with PD-1/PD-L1 blockage. Moreover, patients with low-expression of DLX were observed to have low TIDE prediction scores (*p* < 0.0001), indicating better ICI treatment response. (Figure 6(c)).

Based on data of 9 LUSC patients receiving nivolumab monotherapy in GSE126045 dataset, DLX2-low group was associated with a numerically prolonged PFS (Figures 6(d), 3.4 months [95% CI 0-9.20] vs 1.1 months [95% CI 0-2.86]; *p* = 0.866). Though the statistically significant difference was not reached, the tendency was observed. Based on clinical data of 348 patients receiving atezolizumab in IMvigor210 dataset, patients in DLX2-low group have significant longer OS than those in DLX2-high group (median OS: 10.8 vs 7.4 months; hazard ratio [HR]=0.74, 95%CI 0.57-0.96; *p* = 0.024) (Figure 6(e)).

## 4. Discussion

In this study, we aimed to determine immune-related genes which have potential efficacy predicting value of immunotherapy in patients with LUSC. Data of TCGA-LUSC was applied to discover immune-related genes, three GEO datasets were used for validation, besides IMvigor210 dataset of 348 patients with urothelial carcinoma receiving the treatment of anti-PD-L1 antibody atezolizumab and GSE126045 dataset of 9 patients with LUSC under nivolumab treatment were utilized for identifying the prognostic value of DLX2 on ICI treatment. The integrative results of a series of integrative bioinformatics analysis revealed for the first time that DLX2 was a potential immune-related gene, and low expression of DLX2 might predict a more active immune environment and better response to ICIs treatment in LUSC patients.

DLX gene family is composed of six members DLX1/2, DLX3/4, and DLX5/6 genes which serve as bigene clusters in the genome on different chromosomes, act as transcriptional factor and have indispensable roles in embryonic morphogenesis and postnatal development [26, 27]. In human, DLX2 is located on chromosome 2q31.1. Acting as a transcriptional activator, DLX2 was found to play a role in terminal differentiation of interneurons, it also might play a role in patterning and morphogenesis of craniofacial region [28–30]. While several researches have proposed the association between dysregulation of DLX2 and both solid and hematological malignancies. The overexpression of DLX2 was related to poor prognosis of hepatocellular carcinoma, glioblastoma, etc [31, 32]. It was found to promote the proliferation and metastasis of prostate cancers [33]. In a study of gastric cancer, increased expression of DLX2 was found to be correlated with more advanced stage, but it was not an independent prognostic factor [34]. In basic research, DLX2 was found to have correlation with radiation-induced epithelial-mesenchymal transition and resistance of radiotherapy [35].

While its value in LUSC has not been explored previously. In this study, the expression of DLX2 was firstly identified as a candidate prognostic factor specifically on patients receiving immunotherapy. Additionally, analyses on clinicopathological characteristics of DLX2 revealed that its expression remained stable as age, gender, or TNM stage change; we can speculate that its function might remain consistent in different subgroups of LUSC patients. The results of function enrichment analysis showed that low-expression of DLX2 was closely related to various immune-related pathways like T/B/NK cell mediated immunity, interferon gamma/alpha response, and various autoimmune diseases. Interferon-*γ* (IFN-*γ*)/Interferon-*α* (IFN-*α*) response was found to positively relate to low expression of DLX2. IFN-*γ* is a kind of cytokine produced predominantly by T cells and NK cells in response to a variety of inflammatory or immune stimuli [36]. IFN-*γ* has been identified as an irreplaceable role in the activation of cellular immunity and the stimulation of antitumor immune response [37]. The treatment of ICIs would lead to the production of IFN-*γ*, then promote the elimination of cancer cells [38]. Besides high expression of IFN-*γ* was shown accompanying with significantly longer PFS in patients with NSCLC or melanoma patients [39]. While the role of IFN-*γ* is controversial since studies have also proposed its relationship with tumor progression, which needs further investigation [37]. Additionally, IFN-*α* was found to augment the expression of PD-L1 in immune cells, which can also explain the better outcome of PD-1 inhibition in subgroup with low expression of DLX2 [40].

Low-expression DLX2 is related with various autoimmune disease (AID) like SLE and autoimmune thyroid disease as presented in KEGG functional enrichment analysis. Autoantibodies of AIDs might enhance the presentation of cancer antigens to immune cells, improving immune surveillance. Though the correlation between elevated autoantibody levels and higher incidence of immune-related adverse events (irAEs) has been proposed in previous studies [41, 42]. Its correlation with better clinical outcome of immune-related disease has also been observed. The result of a cohort of 137 patients with advanced NSCLC who received nivolumab or pembrolizumab monotherapy demonstrated that PFS was significantly prolonged in patients with any preexisting autoimmune antibodies like rheumatoid factor, antinuclear antibody, antithyroglobulin, and antithyroid peroxidase than those without (6.5 months vs. 3.5 months, HR = 0.53, *p* = 0.002) [42].

LUSC has been found to have more predicted neoepitopes than lung adenocarcinoma [43]. Besides, alternations of many pathways associated with development and progression of malignancies (such as Notch, Hedgehog, Wnt, and ErbB pathways) were found to significantly overexpress in LUSC compared to LUAD [44]. Function enrichment analysis also revealed that higher expression of DLX2 was associated with cell growth and development, Wnt signaling pathway, histone methylation, transforming growth factor *β* (TGF-*β*) pathway, and many cancers. TGF-*β* has been established to play roles in cancer growth, differentiation, migration, and progression [45]. Recently, TGF-*β* was found to suppressed T helper 2-cell-mediated cancer immunity, targeting TGF-*β* signaling blockade in CD4^+^ T cells is a novel way to remodel TME and restrict tumor progression [46–48]. Some epigenetic aberrations such as DNA methylation, histone acetylation, or methylation were found to help tumor cells to escape immune surveillance. Increased immune- or inflammatory-related gene signatures were observed when inhibiting epigenetic mechanisms [49]. Inhibition of histone methylation is a way to promote tumor immunogenicity and improve the effectiveness of immunotherapies when combined with ICIs [50, 51].

In the analysis of TME, low expression of DLX2 was associated with various tumor-infiltrating immune-positive cells like activated CD4/CD8 T cells, memory CD4/CD8 T cells, M1, etc. Research has found activation of Wnt signaling pathway suppressed the proliferation of CD8^+^ memory T cells and differentiation of effector T cell, generated CD8^+^ memory stem cells and enhanced the polyfunctionality of memory CD8^+^ T cell [52–54]. DLX2 functions as a transcription factor of Wnt signaling pathway, which can partly explain the more abundant infiltration of immune-enhancing cells in DLX2 low expression group. Patients with low expression of DLX2 was also found to have higher M1 macrophage counts. M1 macrophages play an important role in immune function by directly mediating cytotoxicity and antibody-dependent cell-mediated cytotoxicity (ADCC) to kill tumor cells [55, 56].

Limitations existed in this study. Firstly, since limited number of LUSC patients with ICI treatment was available to confirm the prognostic value of DLX2, its significant significance might be ignored, while the better ICI treatment outcome in DLX2-low patients was observed in a large sample-size of patients with other cancers. Larger population-based studies are warranted to identify its prognostic value on ICI treatment of LUSC patients. Secondly, this study is only based on integrated bioinformatical analysis, experimental evidence should be added in future research to support the results of this study.

## 5. Conclusion

Our study of integrated bioinformatical analysis implied that DLX2 could serve as a promising indicator for remodeling TME status and predicting the immunotherapy treatment outcome of LUSC. Further investigations are warranted to explore the potential prognostic value of DLX2 on ICI treatment and the underlying mechanisms.

## Data Availability

The datasets in this study were downloaded from the TCGA database (https://portal.gdc.cancer.gov), GEO database (https://www.ncbi.nlm.nih.gov/geo), and IMvigor210CoreBiologies R package.

## Supplementary Materials

Supplementary 1 Supplementary figure 1. Correlation of DLX2 expression and clinicopathological characteristics of TCGA-LUSC. Age (a), gender (b), TNM stage (c), T stage (d), N stage (e), and M stage (f). Supplementary figure 2. Functional enrichment analysis of DLX2 by GSVA. (a) GOBP enrichment analysis. (b) Hallmark enrichment analysis. (c) KEGG enrichment analysis.

Supplementary 2 Supplementary Table 1. Datasets used in this study. Supplementary Table 2. Four pathways with key genes in Molecular Signatures Database. Supplementary Table 3. The clinicopathological characteristics of TCGA-LUSC. Supplementary Table 4. The differential analysis results of five DEGs in the four pathways.
