# Supplementary material for: DLX2 Is a Potential Immune-Related Prognostic Indicator Associated with Remodeling of Tumor Microenvironment in Lung Squamous Cell Carcinoma: An Integrated Bioinformatical Analysis
Source: Dis Markers. 2022 Oct 21;2022:6512300. doi: 10.1155/2022/6512300 (PMC9617027; doi:10.1155/2022/6512300)
Supplement: Supplementary 1 — Supplementary figure 1. Correlation of DLX2 expression and clinicopathological characteristics of TCGA-LUSC. Age (a), gender (b), TNM stage (c), T stage (d), N stage (e), and M stage (f). Supplementary figure 2. Functional enrichment analysis of DLX2 by GSVA. (a) GOBP enrichment analysis. (b) Hallmark enrichment analysis. (c) KEGG enrichment analysis. [file 6512300.f1.docx]

**Supplementary figure 1**. Correlation of DLX2 expression and clinicopathological characteristics of TCGA-LUSC. Age (A), gender (B), TNM stage (C), T stage (D), N stage (E) and M stage (F).

**Supplementary figure 2**. Functional enrichment analysis of DLX2 by GSVA. (A) GOBP enrichment analysis. (B) hallmark enrichment analysis. (C) KEGG enrichment analysis.
