# Supplementary material for: DLX2 Is a Potential Immune-Related Prognostic Indicator Associated with Remodeling of Tumor Microenvironment in Lung Squamous Cell Carcinoma: An Integrated Bioinformatical Analysis
Source: Dis Markers. 2022 Oct 21;2022:6512300. doi: 10.1155/2022/6512300 (PMC9617027; doi:10.1155/2022/6512300)
Supplement: Supplementary 2 — Supplementary Table 1. Datasets used in this study. Supplementary Table 2. Four pathways with key genes in Molecular Signatures Database. Supplementary Table 3. The clinicopathological characteristics of TCGA-LUSC. Supplementary Table 4. The differential analysis results of five DEGs in the four pathways. [file 6512300.f2.docx]

**Supplementary Table 1. Datasets used in this study.**

| Dataset | Samples | Data type | | description | URL |
| --- | --- | --- | --- | --- | --- |
| TCGA-LUSC | LUSC (n=482) | RNA-seq | Discovery dataset | | https://cancergenome.nih.gov/ |
| GSE157009 | LUSC (n=249) | Affymetrix HG-U133+2 (GPL570) | Validating dataset | | [https://www.ncbi.nlm.nih.gov/geo/query/acc.cgi?acc=GSE157009](https://www.ncbi.nlm.nih.gov/geo/query/acc.cgi?acc=GSE157009) |
| GSE157010 | LUSC (n=235) | Affymetrix HG-U133+2 (GPL570) | Validating dataset | | [https://www.ncbi.nlm.nih.gov/geo/query/acc.cgi?acc=GSE157010](https://www.ncbi.nlm.nih.gov/geo/query/acc.cgi?acc=GSE157010) |
| GSE19188 | LUSC (n=27) | Affymetrix HG-U133+2 (GPL570) | Validating dataset | | [https://www.ncbi.nlm.nih.gov/geo/query/acc.cgi?acc=GSE19188](https://www.ncbi.nlm.nih.gov/geo/query/acc.cgi?acc=GSE19188) |
| GSE126045 | LUSC receiving anti-PD-1 therapy (n=9) | RNA-seq | Validating dataset | | [https://www.ncbi.nlm.nih.gov/geo/query/acc.cgi?acc=GSE126045](https://www.ncbi.nlm.nih.gov/geo/query/acc.cgi?acc=GSE126044) |
| IMvigor210 | Urothelial cancer receiving anti-PD-L1 therapy (n=348) | RNA-seq | Validating dataset | | “IMvigor210CoreBiologies” R package |

PD-1: programmed cell death protein 1; PD-L1:programmed cell death-ligand 1.

**Supplementary Table 2. Four pathways with key genes in Molecular Signatures Database**

| 1 | GOBP_CD8_POSITIVE_ALPHA_BETA_T_CELL_ACTIVATION <http://www.gsea-msigdb.org/gsea/msigdb/cards/GOBP_CD8_POSITIVE_ALPHA_BETA_T_CELL_ACTIVATION> LILRB1 LILRB4 PSMB11 PTPN22 CD274 NCKAP1L HFE HLA-A HLA-E IRF1 PAX1 CLEC4A ZBTB7B CRTAM SH3RF1 WDFY4 BCL2 XCL1 VSIR EOMES RUNX1 RUNX3 CBFB SOCS1 TNFSF8 MAPK8IP1 TOX |
| --- | --- |
| 2 | GOBP_CD8_POSITIVE_ALPHA_BETA_T_CELL_DIFFERENTIATION <http://www.gsea-msigdb.org/gsea/msigdb/cards/GOBP_CD8_POSITIVE_ALPHA_BETA_T_CELL_DIFFERENTIATION> LILRB4 PSMB11 NCKAP1L IRF1 PAX1 ZBTB7B BCL2 EOMES RUNX1 RUNX3 CBFB SOCS1 TNFSF8 TOX |
| 3 | GOBP_CD8_POSITIVE_ALPHA_BETA_T_CELL_PROLIFERATION <http://www.gsea-msigdb.org/gsea/msigdb/cards/GOBP_CD8_POSITIVE_ALPHA_BETA_T_CELL_PROLIFERATION> PTPN22 HLA-A HLA-E IRF1 SH3RF1 XCL1 VSIR MAPK8IP1 |
| 4 | Ferroptosis na ACSL4 AKR1C1 AKR1C2 AKR1C3 ALOX15 ALOX5 ALOX12 ATP5MC3 CARS1 CBS CD44 CHAC1 CISD1 CS DPP4 FANCD2 GCLC GCLM GLS2 GPX4 GSS HMGCR HSPB1 CRYAB LPCAT3 MT1G NCOA4 PTGS2 RPL8 SAT1 SLC7A11 FDFT1 TFRC TP53 EMC2 AIFM2 PHKG2 HSBP1 ACO1 FTH1 STEAP3 NFS1 ACSL3 ACACA PEBP1 ZEB1 SQLE FADS2 NFE2L2 KEAP1 NQO1 NOX1 ABCC1 SLC1A5 GOT1 G6PD PGD IREB2 HMOX1 ACSF2 |

**Supplementary Table 3: The clinicopathological characteristics of TCGA-LUSC.**

| Clinic–pathological features |  | Total (N=482) |
| --- | --- | --- |
| Age |  |  |
|  | <=65 | 185 (38.4%) |
|  | >65 | 291 (60.4%) |
|  | Unknown | 6 (1.2%) |
| Gender |  |  |
|  | Female | 125 (25.9%) |
|  | Male | 357 (74.1%) |
| TNM stage |  |  |
|  | I | 236 (49.0%) |
|  | II | 154 (32.0%) |
|  | III | 81 (16.8%) |
|  | IV | 7 (1.5%) |
|  | Unknown | 4 (0.8%) |
| T stage |  |  |
|  | T1 | 111 (23.0%) |
|  | T2 | 282 (58.5%) |
|  | T3 | 67 (13.9%) |
|  | T4 | 22 (4.6%) |
| N stage |  |  |
|  | N0 | 306 (63.5%) |
|  | N1 | 125 (25.9%) |
|  | N2 | 40 (8.3%) |
|  | N3 | 5 (1.0%) |
|  | Unknown | 6 (1.2%) |
| M stage |  |  |
|  | M0 | 396 (82.2%) |
|  | M1 | 7 (1.5%) |
|  | Unknown | 79 (16.4%) |

**Supplementary Table 4. The differential analysis results of five DEGs in the four pathways.**

| **Pathways** | **gene** | **log FC** | **p value** | **FDR** |
| --- | --- | --- | --- | --- |
| CD8+ αβT cell activation | DLX2 | -1.74124515 | 2.09E-07 | 9.34E-07 |
|  | ERAS | -2.742450291 | 3.58E-06 | 1.28E-05 |
|  | SELENOV | -1.661777165 | 3.52E-05 | 1.02E-04 |
|  | UPK1A | -2.57471448 | 2.00E-04 | 4.98E-04 |
|  | ACTL6B | -4.115132856 | 2.46E-07 | 1.09E-06 |
| CD8+ αβT cell differentiation | DLX2 | -1.500585504 | 3.48E-04 | 0.001270941 |
|  | ERAS | -1.373310325 | 0.006118153 | 0.015113967 |
|  | SELENOV | -1.035219309 | 0.019520859 | 0.040212275 |
|  | UPK1A | -2.074394631 | 0.002431468 | 0.006929334 |
|  | ACTL6B | -3.795185709 | 9.10E-05 | 3.83E-04 |
| CD8+ αβT cell proliferation | DLX2 | -1.484191481 | 4.69E-06 | 1.53E-05 |
|  | ERAS | -2.636950476 | 6.92E-05 | 1.79E-04 |
|  | SELENOV | -1.52629699 | 1.44E-06 | 5.17E-06 |
|  | UPK1A | -2.709787272 | 5.76E-05 | 1.51E-04 |
|  | ACTL6B | -3.858796158 | 1.56E-05 | 4.58E-05 |
| Ferroptosis | DLX2 | -1.605556031 | 0.002568211 | 0.00443619 |
|  | ERAS | -1.343535778 | 0.010952629 | 0.016736721 |
|  | SELENOV | -1.155300366 | 0.009522196 | 0.014720056 |
|  | UPK1A | -2.742825928 | 1.75E-04 | 3.77E-04 |
|  | ACTL6B | -2.373610654 | 0.008203979 | 0.012848968 |

DEGs, differentially expressed genes.
